# Successful Models of Virtual Experiential Education Initiatives in Global Health for International Students

**DOI:** 10.5334/aogh.4547

**Published:** 2024-11-27

**Authors:** Mellissa Withers, Shubha Kumar, Vivian Lee, Indri Hapsari Susilowati, Catherine Zhou, Leander Penaso Marquez, Eleanor Vandegrift

**Affiliations:** 1 Keck School of Medicine, University of Southern California, Los Angeles, CA, USA; 2 Center for Learning Enhancement and Research, The Chinese University of Hong Kong, Hong Kong; 3 Faculty of Public Health, Universitas Indonesia, Indonesia; 4 Office of the Dean of Engineering, The Hong Kong University of Science and Technology, Hong Kong; 5 College of Education, University of the Philippines Diliman, Philippines; 6 ISIP Pinas, Philippines; 7 PAVESOC, Inc, Philippines; 8 Global Studies Institute, University of Oregon, Eugene, OR, USA

**Keywords:** Internationalization, Asia-Pacific, education, global health, experiential learning

## Abstract

*Background:* Experiential learning activities help students prepare for their future careers by providing opportunities for hands‑on practice experiencing real‑world scenarios. Innovations in technology can facilitate experiential learning and cross‑cultural connections for large groups of students in multiple global settings through a virtual platform. However, designing these opportunities with diverse groups of students for a virtual environment can be challenging.

*Objective:* The purpose of this paper is to highlight three examples of innovative virtual experiential learning initiatives that were developed and implemented by the Global Health Program of the Association of Pacific Rim Universities (APRU), a non‑profit network of 60+ leading research universities in the Asia‑Pacific.

*Findings:* We have leveraged the expertise of our wide network to enhance student learning through the purposeful design of virtual educational experiences centered around pedagogical approaches that emphasize active learning, self‑reflection, and knowledge exchange with people from other cultures and disciplines. The annual global health joint virtual courses, the annual APRU Virtual Global Health Case Competition, and the APRU Mini Certificate foster meaningful engagement with other students and experts in the field, expanding the lens of students to foster an increased awareness and appreciation of the diversity of perspectives represented in an international network such as APRU. These allow students to practice real‑world application of knowledge gained in a traditional didactic classroom setting.

*Conclusions:* The benefits of virtual experiential learning to students greatly outweigh the challenges in the design and implementation of such programs. While relatively short‑term, these virtual initiatives have had a demonstrable impact on student participants. Such programs can enhance student learning and provide cost‑effective ways to allow large international cohorts to experience global experiential learning.

## Introduction

Internationalization of higher education has been defined as “the process of integrating an international, intercultural, or global dimension into the purpose, functions, or delivery of post‑secondary education [1].” The importance of internationalization has been highlighted in higher education institutions globally. For universities, major benefits of internationalization can include the establishment of valuable international networks to promote collaborative research, revenue generation through student exchanges, improved global rankings based on internationalization metrics, and building associations that advance policy and advocacy around issues facing higher education [2]. Hosting international students can also improve the overall quality of education offered through diversifying the learning environment for domestic students. For students, internationalization also offers a multitude of benefits, including the development of skills such as leadership, foreign language, and adaptability, which allow students to be more competitive in their future careers. In addition, such cultural experiences help students to become respectful global citizens by building cross‑cultural sensitivity and respect for others.

Internationalization traditionally has focused on student academic exchanges with institutions in other countries [3–5]. Study abroad or international work placements are resource‑heavy, with complex issues to navigate, including accessibility for less financially privileged students [6]. Recent trends have included internationalization at home, defined as the “purposeful integration of international and intercultural dimensions into the formal and informal curriculum for all students, within domestic learning environments” [7, 8]. Innovations in technology and teaching methodologies have dramatically transformed the virtual educational landscape and can facilitate experiential learning with cross‑cultural connections through a virtual platform [9–12].

Experiential learning activities help students prepare for their future careers by providing opportunities for hands‑on practice of real‑world scenarios [13–15]. These opportunities can challenge students to go beyond their comfort zones through interactions and collaboration with peers, which allows them to build professional skills, behaviors, and identities [16, 17]. However, designing effective experiential learning opportunities with diverse groups of students for a virtual environment can be challenging [11]. Mittelmeimer et al. (2023) highlighted the importance of purposefully designing structured active learning for intercultural cohorts to include regular reflective activities and pedagogies that value cultural knowledge sharing [6].

The purpose of this paper is to highlight three examples of innovative virtual experiential learning initiatives developed and implemented by the Global Health Program of the Association of Pacific Rim Universities (APRU), a non‑profit network of 60+ leading research universities in the Asia‑Pacific [18]. The APRU Global Health Program aims to expand existing collaborative research efforts among Pacific Rim universities to address regional and global health issues. Through the Program, APRU members leverage their leading‐edge research, education, and training programs and evidence‐based health interventions to address today’s most pressing challenges. Since its launch in 2007–2008, the program has covered a significant range of topics including existing and emerging public health diseases, migration, mental health, climate change, nutrition, and increasingly urbanized and older populations. Such issues are often recognized as beyond the capacity of individual economies to address alone. Global health recognizes that health is determined by conditions, issues, and concerns that typically transcend national boundaries [19]. The examples outlined in this paper were conceptualized as ways to provide interaction and exchange around these global challenges for large groups of students from many different economies at the same time in a virtual setting.

## Findings

### Distance education global health courses

First, we offer two virtual semester‑long, graduate‑level courses, each of which has been offered annually for at least nine years—one on global health leadership and one on global health ethics. The course model was co‑developed by multiple APRU universities and is managed by the APRU Global Health Program. In the model, a lead university is responsible for developing the online course content (such as a syllabus, readings, assignments, and discussion prompts) and hosting the shared course content on a learning management system that can be accessible by other university partners, such as Canvas. Joint sessions last about two hours each and occur over a ten‑week period. Students sign on simultaneously once a week for live lectures and discussion sessions (via zoom). The first hour of the session consists of live guest lectures by renowned global health leaders and practitioners from various agencies around the globe, including those working at the World Health Organization and other United Nations (UN) agencies, country governments, non‑governmental organizations, and more. This offers a unique learning opportunity to hear from experts, who are often not in reach of a single faculty or university alone. The second hour typically consists of students participating in virtual breakout rooms for small group presentations and discussions about real‑world case studies (including from textbooks that have been developed by APRU partners). Students teamed up with students from each of the other universities and meet outside of class time to discuss group projects and assignments. The shared course website also incorporates assignments such as professional development and self‑reflective exercises, as well as shared discussion boards on takeaway points from speakers and prompts on relevant topics from the courses.

The topics in both courses are designed to address diversity and inclusion, social justice, and global citizenship. For example, the course on global health leadership regularly features speakers on Indigenous rights, climate change justice, mental health, gender equity, and disability rights. The course on global health ethics fosters dialog on controversial topics such as transgender athletes, human genome editing, medical conscientious objection, and commercial surrogacy.

Each fall, four to six APRU universities in different economies participate and students obtain course credit through their own institutions. Typically, 60–100 students enroll in each course and are supported by six to ten instructors and teaching assistants across the participating universities. Over ten years, a total of 21 universities in 11 economies participated in the two courses, with more than 1,220 students enrolled (see Tables 1 and 2). In 2023, students represented 12 academic disciplines in the global health ethics course and up to 20 different disciplines have been represented in this course in previous terms.

**Table 1:** Global Health Leadership (*n* = 619 students, 12 universities).

YEAR	TOTAL NO. OF STUDENTS	TOTAL NO. OF PARTICIPATING UNIVERSITIES	PARTICIPATING ECONOMIES
2015	39	5	Hong Kong, Japan, Taiwan, USA
2016	34	4	Japan, Taiwan, USA
2017	35	4	Japan, Taiwan, USA
2018	n/a	n/a	
2019	67	5	China, Japan, Malaysia, USA
2020	93	7	China, Japan, Malaysia, Mexico, Singapore, USA
2021	82	7	China, Japan, Mexico, Singapore, USA
2022	86	7	China, Mexico, Philippines, Singapore, Thailand, USA
2023	99	6	China, Mexico, Singapore, Thailand, USA
2024	84	6	China, Mexico, Singapore, Thailand, USA

**Table 2:** Global health ethics (*n* = 608 students, 9 universities).

YEAR	TOTAL NO. OF STUDENTS	TOTAL NO. OF PARTICIPATING UNIVERSITIES	PARTICIPATING ECONOMIES
2016	63	6	Japan, Hong Kong, Mexico, the Philippines, USA
2017	41	4	Hong Kong, Mexico, the Philippines, USA
2018	58	4	Hong Kong, Mexico, the Philippines, USA
2019	64	4	Hong Kong, Mexico, the Philippines, USA
2020	84	4	Hong Kong, Mexico, the Philippines, USA
2021	65	5	Japan, Hong Kong, Mexico, the Philippines, USA
2022	76	4	Japan, Hong Kong, Mexico, USA
2023	81	5	Ecuador, Hong Kong, Malaysia, Mexico, the Philippines, USA
2024	76	5	Ecuador, Hong Kong, Mexico, USA

The diversity of students and economies represented in the courses allows for a rich discussion of the course topics including consideration of differing perspectives, fostering intercultural learning. Students from different disciplines learn how to work together on one problem, which is essential to such interdisciplinary subjects as global health. At these courses students also have a lot of opportunities to practice cultural competency skills and cross‑cultural communication with peers across economies and regions. All of these are key competencies for global health students, future leaders, and experts in a variety of fields that can contribute to global health, such as law, public policy, and technology.

An online survey conducted with participants of the 2023 Global Health Leadership course (*n* = 85) revealed that the students identified that the course most helped them develop both personally and professionally in many ways. This included developing or honing skills in the following areas: appreciation of others’ viewpoints and opinions, global collaboration, ability to work in a team, cultural sensitivity/humility, and an appreciation for diversity, equity and inclusion. About 96% of students responded that they would recommend this course to other students.

### Virtual global health case competition

Case competitions are a popular educational tool to promote global health inquiry‑based learning and help students hone their problem‑solving and teamwork skills, skills that employers value because they prepare new graduates to join the workforce [20–24].

The annual APRU Virtual Global Health Case Competition promotes critical thinking on real‑world global health challenges. It also fosters scholastic and digital creativity, team‑work, and problem‑solving skills. In this competition, teams have 10–12 weeks to prepare a program proposal in response to a pressing and timely global health issue. They are required to submit a ten‑minute video in English outlining their solution to the challenge prompts, such as program objectives, specific activities, evaluation plan, budget, and timeline.

The topic of each year’s challenge is collaboratively decided by members of the APRU Global Health Advisory Group and the host university of the annual conference. An outside partner is then identified to provide technical assistance in conceptualizing the challenge parameters. Previous partners have included the United Nations Development Programme (UNDP), the United Nations High Commissioner for Refugees (UNHCR), Amazon Web Services, the Pacific Urban Resilience Lab, and the Diabetes Association of Thailand. The case itself is written by a small team from the APRU Global Health Program and approved by the Advisory Group and the outside partner.

The videos are judged by a team of global health experts from around the world identified through the APRU network and the outside partner agency. In the first round, each video is scored by two judges from two different economies. The top ten videos go to a second round of judging, in which they are scored by different judges, who select the top three finalists. The finalist videos are screened at the annual APRU Global Health Conference, and the first place team wins a prize of US$1,000. Feedback from the judges is shared with the teams. The videos are posted on a publicly available website and the links are shared with all of the teams so that all participants can view the proposals of the finalists and learn from the other teams. Examples of previous years’ participation and topics are shown in Table 3.

**Table 3:** List of case competition topics and participants.

YEAR	TITLE	TEAMS	ECONOMIES	UNIVERSITIES	WINNER
2016	Preparing Pacific Rim countries for natural disasters	13	8	12	Our Lady of Fatima University (Philippines)
2017	Promoting smoke‑free campuses in the Pacific Rim	38	10	19	Universitas Indonesia (Indonesia)
2018	Reducing mental health stigma among university students	27	9	13	University of Southern California (USA)
2019	Social networking to promote physical activity among young people in urban environments	45	10	19	University of Hong Kong (Hong Kong)
2020	Improving elder care in the Asia‑Pacific	45	12	22	Universitas Indonesia (Indonesia)
2021	Technology‑driven solutions to the COVID‑19 infodemic	121	17	36	University of the Philippines (Philippines)
2022	Pandemic preparedness for vulnerable populations in Fiji	48	12	22	Chinese University of Hong Kong (Hong Kong)
2023	Bridging barriers to care: Reaching urban migrant communities	49	12	19	Chinese University of Hong Kong (Hong Kong)
2024	Improving diabetes care management through technological innovation	108	14	31	Universitas Indonesia

Since 2016, the competition has grown from ten participating teams to 108 teams in 2024, with a record of 120 teams in 2021. Overall, an average of 55 teams compete in this international competition each year. Teams must comprise three to six students and can be undergraduate or graduate students or a combination of both. To date, over 2,200 students from 51 universities in 40 economies have participated in the competition. Students can also come from any discipline, although previous student participation is heavily skewed toward medical and public health students. For example, in 2024, 35 disciplines were represented, including fields like bioengineering, environmental health, public health, medicine, economics, information systems, biological sciences, finance, business, nutrition science, physics, biochemistry, dentistry, accounting, physical therapy, veterinary science, pharmacy, occupational health, sociology, psychology, computer science, and more.

### Mini certificate in health research ethics

Training on how to conduct ethical research with human subjects is a crucial part of global health education. However, many universities do not provide specific training in health research ethics for students, despite the requirement to complete a final research project as part of their degree programs. Consequently, students lack information about the main principles of ethics to guide their research and ensure protection of research participants. In order to address this gap, the APRU Global Health on Bioethics Working Group created a free virtual mini‑certificate in health research ethics to strengthen infrastructure to support ethics training in health research. The overall goal is to provide an interactive, interdisciplinary virtual health research ethics training for students at APRU member institutions and other outside institutions to build student capacity for ethical research. A group of seven bioethicists, social scientists, lawyers, and other global health practitioners collaborated to decide on the model and develop the content.

The Mini Certificate in Health Research Ethics has been offered seven times between 2021 and 2024. The training, totaling 12 hours, is designed for students interested in health research across many disciplines. Students join three sessions of two hours, 15 minutes each, plus complete self‑paced asynchronous homework available on a shared course website. The training employs a practical approach to learning about ethics through short live guest lectures and analysis of real‑world case studies. In each session, two expert speakers deliver short talks of 30 minutes on the basic principles of ethics, including beneficence, justice, respect for persons, community‑based research, and the function of institutional review boards, also known as research ethics boards. The shared course website also provides background reading, discussion boards to further the discussions and case studies, self‑reflective exercises, and other resources.

Faculty select case studies that will provide opportunities for students to concretize key concepts, review previous topics, and practice solving case studies. Each breakout room has an expert facilitator who reviews the cases and guides the discussions. During each session, students are assigned to different breakout rooms, to ensure they have the opportunity to interact with students and expert facilitators from across the APRU network. Both the students and facilitators came from diverse disciplines, cultures, and research backgrounds.

The facilitators are provided with a facilitator guide with tips on how to promote active learning in this environment. This includes suggestions for encouraging participation from all students and handling challenging conversations. A one‑hour facilitator training workshop is also provided to new facilitators.

Between 25 and 40 facilitators from at least ten Asia‑Pacific economies participate in each training session. In addition to training students, the program has also built capacity among faculty and researchers within the APRU network to be able to train students in health research ethics through the set of core curricula and resources provided to facilitators. Further, about 10% of spots in each training have been reserved for non‑students, including faculty, researchers, and members of ethics review boards.

Generally, 200–250 students, both undergraduate and graduate students, are accepted for each session, with priority given to students from APRU member institutions. However, about 10% of spots are given to students from universities in low‑ or middle‑income economies that are not part of the APRU network. To ensure a diverse group of students in each session, quotas are used for each economy, so that no more than 30 students are accepted from any single economy.

To date, over 1,000 students have completed the training from 55 universities in more than 29 economies in the Asia‑Pacific, as well as South Asia, the Middle East, Europe, Latin America, and Africa. While the majority of students are studying public health and medicine, other fields are commonly represented, including anthropology, biology, business, demography, dentistry, engineering, environmental sciences, food science, indigenous studies, information technology, law, nursing, nutrition, pharmacy, physiotherapy, psychology, and more. The fact that there is usually a waiting list of 200–500 students for each session highlights the value of such training for students.

The student feedback has been overwhelmingly positive. For example, students report that the interactive aspect made the learning experience more dynamic and engaging. In the course evaluation, one student reported that

*These sessions offered an interactive and engaging platform for participants to discuss and exchange ideas actively. It wasn’t just about passively absorbing information; it was about having real conversations with fellow learners. This interactive aspect made the learning experience much more dynamic and allowed me to gain different perspectives on the topics we were covering.*

Students also enjoy the breakout discussions because they are able to build on ideas with feedback and questions from facilitators that provoked critical thinking. Students report that the facilitators helped to keep the group on track, while motivating everyone to speak and contribute their opinions and ideas. One student explained

*We were able to build on ideas with feedback and questions from moderators that provoked critical thinking. In one of my discussion groups where the group found it difficult to start, the moderator guided the discussion by providing options and facilitating critical thinking about choices we opted for.*

In addition, students appreciate the diversity of the students and speakers, which allows them to gain different perspectives and exchange ideas. Many students report that they had taken their own viewpoints for granted but that they have gained a new appreciation for other cultural perspectives from other parts of the world. One student wrote,

*This course has provided me with an opportunity to examine and challenge my own values and beliefs with respect to issues arising from health research ethics. The opportunity to learn from individuals from different countries, as well as different professional backgrounds, further enhanced my ability to view health research ethics issues from a diverse range of perspectives.*

Finally, many students had very favorable views about the practicality of the approach to learning about the ethical principles. One student reported, *“I appreciated the very practical lectures and real‑world scenario, presented as well as the different perspective from my ‘classmates’ all over the world.”*

## Conclusion

We have provided three examples of innovative, long‑term international virtual experiential education in global health. One major objective of these initiatives has been to provide an opportunity for students to solve problems that are relevant in today’s global landscape. By leveraging the APRU Global Health network, each learning experience model was collaboratively developed by a diverse set of experts from multiple economies, thereby enriching the content. Each model was created to maximize the opportunity for students to learn from varied viewpoints and world views, as well as self‑reflection as a key component. We have also paid close attention to ensuring the practical value of the experience for students. Students report that the opportunity to meaningfully engage with students and faculty from other cultures and disciplinary backgrounds was one of the most useful and rewarding aspects of these experiences. They value the opportunity to discuss different worldviews through facilitated dialogues in a safe learning environment. Students also appreciate the practicality of the approaches, using case studies and real‑world scenarios allow students to recognize the application of the topics to their own lives. Further, students report developing communication, leadership, and critical‑thinking skills through these experiences—important skills for solving future global challenges.

Virtual experiential learning allows students to practice real‑world application of knowledge gained in a traditional didactic classroom setting. Providing opportunities for students to interact with students from other economies and disciplinary backgrounds can illustrate the importance of considering multiple perspectives on global health issues, which will be required to solve future global health challenges. The benefits of these programs for students include encouraging personal growth and self‑discovery, developing cross‑cultural communication, and building leadership skills for their future careers. Further, these “internationalization at home” experiences promote inclusion by reducing barriers to participation through global virtual classrooms. We have demonstrated that structured active learning, explicitly designed for intercultural cohorts, can be successfully implemented in a range of national contexts, disciplines, and group sizes.
